# Hypertensive disorders of pregnancy (HDP) management pathways: results of a Delphi survey to contextualise international recommendations for Indonesian primary care settings

**DOI:** 10.1186/s12884-021-03735-3

**Published:** 2021-04-01

**Authors:** Fitriana Murriya Ekawati, Sharon Licqurish, Jane Gunn, Shaun Brennecke, Phyllis Lau

**Affiliations:** 1 Department of Family and Community Medicine, Universitas Gadjah Mada, Sleman, Yogyakarta Indonesia; 2 Department of General Practice, University of Melbourne, Level 2, 780 Elizabeth Street, Melbourne, Victoria 3000 Australia; 3 School of Nursing and Midwifery, Monash University, Clayton, Victoria Australia; 4 University of Melbourne Department of Obstetrics and Gynaecology, Royal Women’s Hospital, Parkville, Victoria Australia; 5 Pregnancy Research Centre, Department of Maternal-Fetal Medicine, Royal Women’s Hospital, Parkville, Victoria Australia

**Keywords:** Delphi survey, Management, Pathways, Hypertensive disorders of pregnancy, Preeclampsia, Indonesia, Primary care

## Abstract

**Background:**

Hypertensive disorders of pregnancy (HDP) are a significant contributor to the high maternal mortality rate in Indonesia. At the moment, limited guidelines are available to assist primary care providers in managing HDP cases. A previous review of 16 international HDP guidelines has identified opportunities for improving HDP management in Indonesian primary care, but it has not determined the suitability of the recommendations in practice. This study aims to achieve consensus among the experts regarding the recommendations suitability and to develop HDP pathways in Indonesian primary care.

**Methods:**

Maternal health experts, including GPs, midwives, nurses, medical specialists and health policy researchers from Indonesia and overseas were recruited for the study. They participated in a consensus development process that applied a mix of quantitative and qualitative questions in three Delphi survey rounds. At the first and second-round survey, the participants were asked to rate their agreement on whether each of 125 statements about HDP and HDP management is appropriate for use in Indonesian primary care settings. The third-round survey presented the drafts of HDP pathways and sought participants’ agreement and further suggestions. The participants’ agreement scores were calculated with a statement needing a minimum of 70% agreement to be included in the HDP pathways. The participants’ responses and suggestions to the free text questions were analysed thematically.

**Results:**

A total of 52 participants were included, with 48, 45 and 37 of them completing the first, second and third round of the survey respectively. Consensus was reached for 115 of the 125 statements on HDP definition, screening, management and long-term follow-up. Agreement scores for the statements ranged from 70.8–100.0%, and potential implementation barriers of the pathways were identified. Drafts of HDP management pathways were also agreed upon and received suggestions from the participants.

**Conclusions:**

Most evidence-based management recommendations achieved consensus and were included in the developed HDP management pathways, which can potentially be implemented in Indonesian settings. Further investigations are needed to explore the acceptability and feasibility of the developed HDP pathways in primary care practice.

**Supplementary Information:**

The online version contains supplementary material available at 10.1186/s12884-021-03735-3.

## Background

Hypertensive disorders of pregnancy (HDP) cover a range of diagnoses, including chronic hypertension, gestational hypertension, white coat hypertension and preeclampsia/eclampsia [1]. Every day, in low-and middle-income countries (LMICs) many women die due to HDP [2] and in Indonesia, HDP remains a major cause of maternal mortality with approximately three maternal deaths daily [3, 4]. With its current increasing trend, HDP in Indonesia may soon replace postpartum hemorrhage as the most common cause of direct maternal mortality [3, 4].

Research shows that maternal mortality from HDP is preventable if the women receive appropriate management. However, many maternal deaths from HDP in Indonesia are not well anticipated due to lack of practice guidelines in primary care [5]. Current Indonesian primary care guidelines merely recommend general practitioners (GPs) to refer women with HDP to hospitals [6–8], but details of further management such as screening, monitoring, and long-term postpartum follow-up treatment for HDP women are lacking [9]. Meanwhile, Indonesian disparities in health due to community beliefs, inadequate obstetrician availability and challenging geographical locations complicate referrals from primary care leading to delays in accessing appropriate hospital management [4, 10, 11]. For these reasons, there is an urgent need to upgrade Indonesian primary care providers’ practice with the aim to prevent severe HDP complications and optimise patient initial treatment before hospitalisation.

This research is part of a larger study to improve the quality of HDP management in Indonesian primary care by developing HDP management pathways appropriate for this settings [12]. The management pathways are designed to enable the providers to have structured, practical and collaborative guidance that subsequently improves their practice behaviour [13–16]. Our previous review has identified evidence-based practices for improving HDP management in Indonesian primary care [17], and our previous interviews with keystakeholders have explored the way HDP is managed in Indonesian primary care [9, 18]. However, to adopt the recommendations to develop HDP management pathway as an intervention to improve the providers’ practice would require a further contextualisation process. Our review findings indicate that not all recommendations based on the international HDP guidelines can directly be adopted into the Indonesian context due to different practice environments, such as different professional authority, facilities, policies or public insurance (*JKN/Jaminan Kesehatan Nasional*) regulations [9, 17].

As well, our interviews with Indonesian key stakeholders in primary care also emphasise a need for further considerations when developing HDP management pathways for Indonesian primary care. Public primary care clinics (Puskesmas/*Pusat Kesehatan Masyarakat*) in Indonesia have to undertake maternal surveillance and maternal audit processes in the community in addition to provide individual patient treatment [9, 18]. There are also community health workers (cadres) involved in the maternal health activities, but their job description in the current Indonesian guidelines remains unclear [19, 20]. Therefore, suggestions arising from the interviews also need to be further explored for their use in the HDP pathways. This research then aims to establish experts’ consensus on the identified 125 international and local HDP management recommendations to develop HDP management pathways for Indonesian primary health care settings.

## Methods

### Study design: consensus development using Delphi technique

Consensus is usually defined as ‘general agreement’ [21]. Consensus development is an essential stage in a guideline or pathway development to determine general experts’ agreement regarding recommendations used in the pathways [21] or to determine the feasibility of international recommendations to be used in a local practice setting where their supporting implementation evidence is limited [22].

One of the most common consensus development methods in pathway development is the Delphi technique, in which the experts provide their judgments directly or indirectly through surveys or interviews [23]. In this study, three rounds of anonymous online Delphi survey were used to develop consensus on the applicability of 125 statements based on international HDP management recommendations and our previous interviews to develop HDP pathways, with respect to their readiness to be used by GPs, midwives and nurses in Indonesian primary care settings [17].

### Survey statements

The HDP statements tested in the survey were informed by our review of international evidence-based HDP guidelines [12, 17] and our exploratory interviews with Indonesian primary care stakeholders [9, 18]. The 125 statements were divided into 62 statements for the first-round survey (topics included in this round: HDP definitions, risks, screening and diagnosis, prevention and long-term follow-up), and 63 statements for the second-round survey (topics included in this round: HDP management, monitoring, facilities and surveillance for women with HDP). The complete survey questionnaires are provided in Supplementary file 1.

### Participants

Indonesian and international experts in maternal health identified through the authors’ professional networks and snowballing recruitment process were invited to participate in the study. The participants’ inclusion criteria were [12]:

(i) GPs, midwives, nurses, specialists, local health officers, maternal health researchers or policymakers.
(ii) Have a minimum of  two-years of research and/or working experience in maternal health;
(iii) Have an academic background in health sciences;
(iv) Familiar with the context of primary care in Indonesia or LMICs; and
(v) Keen to participate in all survey rounds.

### Recruitment

All prospective participants were recruited by email and/or WhatsApp messenger [24] containing a link for the recruitment pages. The recruitment pages were provided bilingually in Bahasa Indonesia and English, and the participants were able to choose their preferred language. Prospective participants who did not meet the inclusive criteria were excluded and only those who satisfied the criteria were able to carry on to the survey’s online plain language statement (PLS) and electronically sign the consent page. The participants’ identity was confidential to other participants and was only be identifiable by researchers in this study.

### Data collection

The data collection applied three rounds of online survey using the University of Melbourne REDCap (Research Electronic Data Capture) platform [25], and each survey round consisted of:

- a questionnaire asking participants to provide their judgement on the HDP statements on a five-point Likert scale [26, 27] (1 = strong disagreement, 2 = disagreement, 3 = indicated neutral position, 4 = agreement, and 5 = strong agreement). Participants were asked to rate their judgment on (i) whether the recommendation was useful to improve HDP management in primary care and (ii) whether the recommendation was likely to be applicable in practice in Indonesia or needed to be contextualised;
- free-text questions to ask the participants further suggestions related to the tested statements.

Participants were given 3 weeks to complete each round. They received short messages and email reminders in 1 week and 3 days before the survey was closed. Once a round was completed, participants who completed it were sent a link to the next survey round.

#### First-round survey

After completing the PLS and consent pages, the participants were sent with the first-round survey link asking them to rate 62 statements: HDP definitions (*n* = 7), risk factors (*n* = 16), screening and diagnosis (n = 16), prevention (*n* = 10), and long-term follow up (*n* = 13) in primary care. Other than the basic free-text questions above, these additional three questions about HDP management in primary care were also presented exclusively in the first-round to gain insights regarding the roles of primary care providers in HDP management [12]:

(i) What are the roles of Indonesian primary care in HDP management?
(ii) What potential practices that can be conducted by primary care providers in HDP management?
(iii) What are the barriers and facilitators of HDP management in Indonesian primary care?

#### Second-round survey

This round tested 63 statements regarding HDP management (*n* = 14), monitoring (*n* = 31), required facilities (*n* = 8), and surveillance (*n* = 10) in primary care. The survey pages also contained results of the first-round survey and participants were able to review and revise their responses on statements that had not reached consensus in the first-round survey.

#### Pathways development

Statements that reached consensus at the first and second-round survey were used to develop HDP pathways that were initially drafted and designed by the first author. However, drafts of the pathways were also discussed and received suggestions from all project investigators before their presentations to participants in the third-round survey.

#### Third-round survey

The third-round survey asked for the participants’ agreement and suggestions on the HDP management pathway drafts. Results of the second-round survey were presented, and the participants were able to review and revise their responses on statements that had not reached consensus in the first and second-round survey. Statements that reached consensus at this round, including the participants’ suggestions were used to finalise the HDP management pathways.

### Data analysis

The participants’ agreement scores in each survey were analysed descriptively using Microsoft Excel software. The participants’ responses were calculated for each statement to generate total agreement scores, standard deviation and interquartile ranges (IQR). IQR was chosen for determining the spread of the data considering that the survey only had five ordinal options (5-point Likert scale) and the limited number of participants in the study [28]. The minimum requirement set for each round was: at least 60% participation and the statements had to have at least 70% agreement to be included in the HDP pathway [23].

The free-text responses and suggestions from the participants were imported into the Nvivo 12 software [29] and analysed thematically [30]. The free-text responses in Bahasa Indonesia had also been translated into English, imported into Nvivo and coded for any significant survey responses. The codes were grouped based on their similarities and patterns, and were then used to establish themes and overarching themes. The themes and overarching themes were also discussed and mutually agreed by all project investigators. Presentation of this study adheres the standard of reporting intervention development studies (GUIDED) (Supplementary file 2) [31].

### Language validation

All of the survey statements and questions were initially created in English. They were then translated into Bahasa Indonesia by the first author and presented to participants based on their language preference. Participants’ responses written in Bahasa Indonesia were also translated into English to enable analysis and discussion between the project investigators. All questionnaires and a quarter of the free-text responses were also back-translated into English and were reviewed by another two native Indonesian speakers to ensure their translation validation.

### Survey validation

The recruitment and survey pages were tested to ensure internal validation. Each of the survey pages had a minimum of ten trials by the project investigators and validation participants before its distribution to the survey participants.

## Results

The surveys were conducted from November 2018 to May 2019. A total of 52 participants agreed to participate, of these, 48 (92.3%), 45 (93.4%), and 37 (82.2%) participants completed the first, second, and third-round of surveys. The participants’ baseline demographic data are presented in Table 1.

**Table 1:** Summary of the participants’ demographic background

Country	
Indonesia	48
International	4
Occupation	
General practitioners	27
Midwives	6
Nurses	6
Obstetricians	4
Cardiologists	2
Policymaker	1
Local health officers	2
Others (emergency medicine doctors, medical consultant)	4
Education background	
Diploma in health sciences	5
Bachelor	14
Master or specialist training	24
Doctoral or sub-specialist	9
Gender	
Man	38
Woman	14
Workplace	
Public primary care clinic	19
Private clinic	9
Public hospital	8
Private hospital	6
Health department	2
University	19
Others	2
Practice experience	
0–5 years	14
6–10 years	17
11–15 years	10
16–20 years	6
> 21 years	5

### First-round survey

Around 85.5% of the tested statements reached consensus at this round and the participants’ agreement scores ranged from 39.6 to 100.0%. Even, some statements achieved 100.0% agreement, such as ‘*routine blood pressure measurement for HDP screening*’ and ‘*pregnant women have to be informed and counseled about HDP risk factors*’. Ten statements did not reach 70.0% agreement and these statements were then brought forward into the second-round survey (Please refer to Table 2 and Suplementary file 3).

**Table 2:** Summary of agreement scores^a^

Recommendations	Agreement score (%)	Quartile 1 (25th percentile)	Median	Quartile 3 (75th percentile)	Interquartile ranges (IQR)	Standard Deviation (±)
**First-round**						
Mean agreement scores on						
Definitions	86.6	4.0	4.0	4.7	0.7	0.8
Risk factors	81.9	3.8	4.0	4.6	0.8	0.8
Screening and diagnosis	81.9	3.7	4.1	4.7	1.0	0.8
Prevention	81.1	3.8	4.0	4.4	0.6	0.8
Long-term follow up in primary care	82.5	3.6	3.9	4.7	1.1	0.7
**Second-round**						
Mean agreement scores on:						
Management	83.0	3.8	4.1	4.9	1.1	0.8
Monitoring	84.1	3.7	4.0	4.6	0.9	0.7
Facilities	96.9	4.0	5.0	5.0	1.0	0.5
Surveillance	98.0	4.0	4.5	5.0	1.0	0.5
**First-round statements that achieved consensus in the second-round**						
High-risk preeclampsia: previous history of systemic lupus erythematosus.	72.9	3.0	4.0	4.3	1.3	0.9
This test is recommended as a baseline reference for women with a high risk of preeclampsia: creatinine	70.8	3.0	4.0	5.0	2.0	1.0
A GP can prescribe low dose aspirin as prophylaxis for preeclampsia.	70.8	3.0	4.0	4.0	1.0	0.9
Aspirin 75-150 mg is given daily at bedtime	70.8	3.0	4.0	4.0	1.0	0.8
**Third-round**						
Mean agreement scores on:						
Hypertensive disorders of pregnancy (HDP) diagnosis flowchart.	86.5	4.0	4.0	4.0	0.0	0.8
HDP management pathways in primary care	83.8	4.0	4.0	5.0	1.0	1.0
Surveillance pathway for women with HDP in primary care.	86.5	4.0	4.0	5.0	1.0	0.8
**First-round statement that achieved consensus in the third-round**						
This test is recommended as a baseline reference for women with high risk of preeclampsia: platelet count	70.8	3.0	4.0	4.0	1.0	1.0

^a^Five-point Likert scale used in the study: 1 = strong disagreement, 2 = disagreement, 3 = indicated neutral position, 4 = agreement, and 5 = strong agreement

Free text-responses in the first-round highlighted the roles of primary care physicians in HDP management, and the most prominent theme emerging in the analysis was authority (with 219 quotes). Most participants believed that primary care providers have responsibilities to conduct antenatal care (ANC), identify women with increased risks of HDP and refer patients who are at risk to obstetricians. They also had to not only provide HDP clinical management in primary care but also care coordination with hospitals.

The participants also conceded that routine ANC had already been well applied in Indonesian primary care practice and blood pressure monitoring and dipstick urine tests been routinely conducted as preeclampsia screening. However, some participants claimed that primary care had limited resources available in practice, for instance, only nifedipine was available as a medicine for pregnancy hypertension and the doctors’ limited time for pregnancy consultation.

Interestingly, many of the clinician participants also indicated their own doubt with the quality of HDP management they currently provide in practice, particularly the referral timing and patient monitoring procedures. They also expected guidance and skills upgrades on such HDP management in primary care (Table 3).

**Table 3:** Quotes from the participants’ free-text questions responses in the survey

First-round survey	*“GPs’ tasks in HDP management include stress management, blood pressure monitoring and management, early detection for haematology, hepatic and renal disorders, and the fetal wellbeing (heartbeat, movement, growth and development). They also assist normal delivery, do postpartum management and make an appropriate, timely, and safe referral if it is needed “(Participant 15, GP).* *“Nifedipine is the only medicine available in primary care for HDP” (Participant’s code 88, GP).* *“ANC time provided for the patients is limited that further limits clinical procedures performed for the patients” (Participant 36, Nurse).* *“More upskill training and standards on HDP management are needed, including screening tools, and further management in primary care. There should also be clear guidance on procedures offered for pregnant women before being referred to the hospitals and0 follow up procedures for women with HDP aiming to reduce HDP impact in the community” (Participant’s code 42, Nurse).*
Second-round survey	*“(Mild) Preeclampsia should be observed for its development to severe preeclampsia. Salt restriction diet is still advised for its potential to increase blood pressure in pregnant women. Methyldopa is also not available in primary care. We only have amlodipine 5 and 10 mg” (Participant 67, GP).* *“It may be useful to involve any policy related to this standard because there is no means of this standard without any endorsing policy from the government” (Participant 85, Midwife).*
Third-round survey	*“The diagnosis pathway is too complicated. It should not be developed through this survey but made by experts with recent evidence” (Participant 62, Obstetrician).* *“You may need to differentiate mild and severe preeclampsia and in obstetrics, we are not familiar with primary or secondary hypertension nor masked and white coat hypertension” (Participant 57, Obstetrician).* *“Management of BP > 140/90 should be under specialist care at the hospitals” (Participant 40, Midwife).* *“Some private practice doctors may not want to have their patients seen in a public clinic if they are a private patient. We also need to ensure patient confidentiality. So, they may not want the community leader involved” (Participant 38, Family doctor).*

### Second-round survey

Most of the tested statements (92.0%) reached consensus in this round. Similar to the first-round survey results, the statements’ agreement scores were high, particularly on statements related to community surveillance and home visits for women with HDP (mean agreement scores: 98.0%). Five statements did not reach consensus in this round and the statements were re-tested in the third-round survey (Please refer to Table 2 and Supplementary file 3).

Seven participants also revised their responses to statements in the first-round survey and increased the agreement scores to above 70.0% for four statements: ‘*systemic lupus erythematosus as a risk factor for preeclampsia*’ (72.9%), ‘*serum creatinine as a baseline examination for women with preeclampsia risk factors*’ (70.8%), ‘*aspirin 75-150mg is given daily at bedtime*’ (70.8%) and ‘*GPs can prescribe low dose aspirin prescription for preeclampsia prophylaxis*’ (70.8%).

Some participants in free-text questions raised further opinions regarding the authority of HDP in primary care, such as suggesting different management of mild and severe preeclampsia based on their usual practice and a need for a government policy to facilitate the pathways’ implementation (Table 3). Again, a participant also wrote another limitation in practice, i.e. that only a few kind of medicines available in primary care.

#### Pathways development

The HDP management pathways drafts had been developed from statements that reached consensus from the first and the second-round survey. HDP management pathways drafts were presented in three flowcharts: (i) HDP diagnosis, (ii) HDP management, and (iii) HDP maternal surveillance flowchart in primary care. The HDP management pathway itself was divided into five sections: (i) screening for preeclampsia risk factors at the first pregnancy visit, (ii) HDP screening activities during routine ANC, (iii) HDP management and monitoring, (iv) delivery plans for women with HDP, and (v) postpartum follow up for women with HDP in primary care.

The project investigators also considered and discussed statements that had not achieved consensus at the first and second-round survey. The pathways accommodated statements related to contraception and antihypertensive medication used for women with HDP history, and were later accommodated using information tables (that are not included in this publication). One statement was also considered not to be re-tested in the third-round survey, i.e., IVF as a risk factor for preeclampsia.

### Third-round

**Fig. 1:**
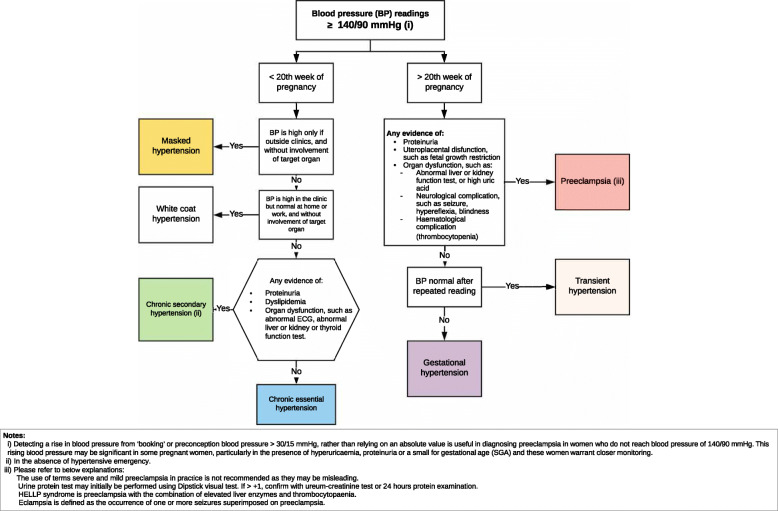
Diagnosis flowchart for women with HDP

Most participants agreed on the HDP management pathway drafts. The pathways’ agreement scores ranged from 78.4% for HDP monitoring to 89.2% for preeclampsia risk factors screening (Table 2 and Supplementary file 3). Eleven participants revised their responses on statements that had not received consensus at the previous rounds. Their revised responses, however, only changed agreement score for platelet count as baseline data for pregnant women with risks of preeclampsia (from 66.7 to 70.8%). The complete final agreement score for each statement and the diagnosis flowchart are attached as Supplementary file 3 and Fig. 1.

There were participants’ suggestions obtained in the third-round survey requesting improvement on the triage for pregnant women by accommodating recommendations for preeclampsia prevention after the 20th week of pregnancy. A further suggestion was also obtained for the HDP surveillance pathway to respect the patients’ confidentiality when sharing their medical information from private to public primary care clinics/Puskesmas. It was previously mentioned at the surveillance pathway draft that any HDP cases should be referred to Puskesmas  to record their surveillance data and be followed-up by cadres, such as by doing home visits or receiving supports from community leaders. The suggestion for patient confidentiality was then used to improve statements listed in the surveillance pathway (Table 3).

Another participant in this round also expressed his disagreement on HDP pathway development through the survey. He mentioned that the pathways drafts were way too complicated and they should not be developed through surveys. There were also, again, a suggestion to differentiate management of mild and severe preeclampsia and further pressure to refer women with HDP to hospitals.

#### Final revised pathways

**Fig. 2:**
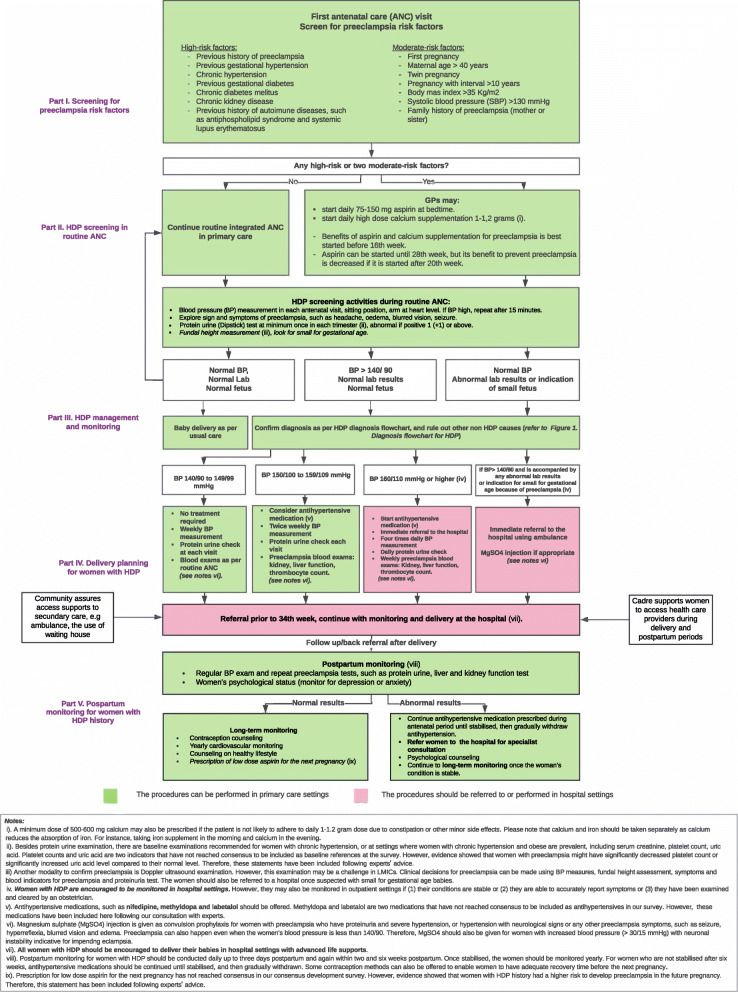
Hypertensive disorders of pregnancy (HDP) management pathway in primary care

**Fig. 3:**
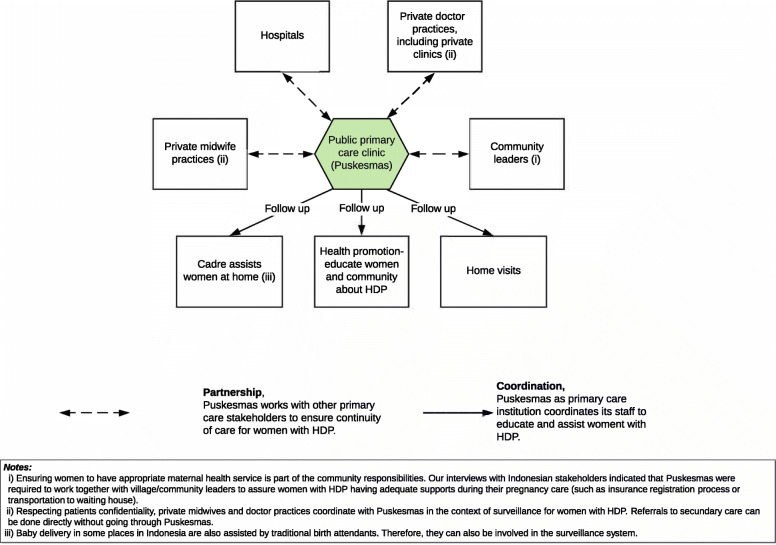
Surveillance pathway for women with hypertensive disorders of pregnancy (HDP) in primary care

The pathways drafts were revised after receiving the participants’ suggestions. The preeclampsia triage during the first antenatal visit was improved by providing statements if aspirin was initiated before the 16th week and after the 20th week of pregnancy. A statement was also included in the HDP surveillance pathway regarding respecting patients’ confidentiality. The participants’ suggestion to differentiate management of mild-moderate and severe preeclampsia was not accommodated in this stage. The final HDP management pathways and surveillance pathways in primary care are presented in Figs. 2 and 3.

## Discussion

This study is the first study to seek experts’ consensus and opinions on a set of HDP management recommendations for the Indonesian primary care setting. Despite some identified challenges that may limit the implementation of the developed pathways in primary care, the surveys demonstrated that almost all of the HDP recommendations and the developed HDP management pathways have reached consensus for their implementation in Indonesia.

he developed pathways provide step-by-step clinical guidance on HDP management embedded in the routine ANC and can shift the clinicians’ focus to early signs, symptoms and risk factors for preeclampsia. The developed pathways also have abilities to equip GPs and midwives in Indonesia with comprehensive HDP guidance in primary care that have been expected by key stakeholders in our exploratory consultation, particularly, when referral to the hospital could not be made immediately [9, 18. The pathways are also able to complement a preeclampsia management model recently developed for LMICs that covers principles of the management but lack of detailed clinical recommendations for primary care [32]. The pathways can also complement other HDP guidelines in Indonesia and other LMICs-which were published more than a decade ago and focused only on preeclampsia management and secondary care [6, 33].

Potential challenges that may limit the recommendations uptakes in practice have also been identified in the survey, such as tensions of interprofessional authority between the clinicians and clinical inertia of HDP management in primary care. It was implied in the survey of the participants’ hesitance to agree on some HDP management conducted by GPs and midwives, such as low-dose aspirin prescriptions in primary care. Even though, aspirin medicine has benefits of reducing risks of preterm preeclampsia [34–36], and relatively safe for pregnant women [37, 38]. Low dose aspirin tablets are also widely available in Indonesian Puskesmas [39]. The participants also seem to resign on the fact that only nifedipine is available in Puskesmas, and hesitate to agree on other antihypertensive prescriptions in primary care, such as methyldopa and labetalol, that are only available in the hospitals or accessible through prescription in private pharmacies [40].

Different practice recommendations based on the existing Indonesian HDP guidelines compared to the international guidelines may also influence the pathways uptake in practice. Some participants recommended different preeclampsia management based on its severity category of mild versus severe preeclampsia. In an Indonesian guideline, pregnant women with blood pressure ≥ 140/90 mmHg and positive (+ 1) proteinuria or increased creatinine level are categorised as having mild to moderate preeclampsia. In comparison, women with severe preeclampsia are those who have blood pressure ≥ 160/90 mmHg, positive (+ 2) proteinuria and/or preeclampsia symptoms such as headache or visual disturbance [8]. However, recent international guidelines on preeclampsia have recommended avoiding those categorisations above in practice, as they are often confusing and that women with preeclampsia can deteriorate very rapidly into more severe conditions [1, 41, 42]. As those categorisations are still common in Indonesian practice, it is therefore not surprising that some participants in the survey suggested formal policy changes to secure practices of additional preeclampsia management in primary care. While some obstetrician participants also voiced their opinions that the pathways should be developed by more competent experts, even though, they have been informed that recommendations in the survey were extracted from international HDP guidelines and backed-up by recent evidence [12, 17].

The clinicians’ hesitance and clinical inertia above are likely influenced by gaps of medical training for GPs and other clinicians in primary care, hierarchical culture and late adoption of evidence-based practices in Indonesian health care. GPs in Indonesia are only required to complete a medical doctor degree in a university to be able to practice in primary care. In contrast, specialists are required to undertake another three to four-years of specialty training at a hospital. This training gap then gives the misconception that GPs are less competent and confident than specialists resulting in the GPs’ low status in the eyes of patients and specialists [43–45], while obstetricians are perceived as having the highest authorities and the ultimate source of maternal care updates.

However, gaps in evidence-based practice above can be improved by introducing and implementing the developed HDP pathways in primary care. The GPs, midwives and nurses in Indonesia have important roles of HDP management due to their gate-keeping roles and accessible practices across Indonesian territory [43]. if they are not well supported and encouraged to perform more HDP management, then who will be able to appropriately manage HDP women in the first place considering challenges of referral and disparities in Indonesian health care.

### Strengths and limitations of the study

Delphi technique in this survey is not bound by geographical locations of the participants and offers flexible opportunities for them to share their opinions and minimising bias of dominant experts [23, 46]. The process is anonymous and hence has the advantage of minimising challenges of the hierarchical culture that we would anticipate among Indonesian health care professionals [47–49].

Although the sample size in this study was small, the recruitment of participants with various backgrounds and experiences has captured broad views and opinions from the experts [50]. The survey has also optimised the participants’ interaction by providing them opportunities to view and revise their responses at the previous rounds. Results validity of the study is justified by the high survey participation rates in each round and the high percentages of agreed statements completed with good agreement scores [23, 51].

There were some statements that were not re-tested in the third-round survey due to local contextual considerations. In-vitro fertilisation (IVF) was not re-tested due to its irrelevance in the Indonesian population context as IVF is usually accessed by subfertile-married couples [52, 53]. Contraception and antihypertensive medication were further accommodated using two tables in the supplementary materials (that are not included in this publication) aiming to provide more comprehensive educational information for the targeted audience in primary care.

#### Suggestion for further research

Further research is desired to investigate the pathways acceptability in practice and to confirm the suitability of aspirin prescription for anemic women. Towards the end of the study, new evidence emerged to indicate that anaemia in pregnancy might also be a risk factor for preeclampsia in LMICs [54, 55]. This possible risk factor is directly relevant to situations in Indonesia where anemia prevalence is high due to malnutrition, genetics, or infections, such as malaria and hookworm, which cause inflammation in the placenta [54–57]. The use of aspirin in anaemic women, however, is not currently supported in any of the existing international HDP guidelines [17] or tested in the survey. Therefore, the management of anaemic women with any preeclampsia risk factors would require further careful clinical consideration.

## Conclusion

Most of HDP management recommendations extracted from international HDP guidelines [17] achieved consensus in this study and the developed HDP management pathways are potentially implementable in Indonesian primary care. Further research is needed to explore the pathways’ acceptability and feasibility in Indonesian practice and to investigate the appropriateness of anemia as another preeclampsia risk factors in LMICs, including the use of low-dose aspirin in anemic pregnant women with other underlying preeclampsia risk factors.

## Supplementary Information

**Additional file 1: Supplementary file 1.** Survey questionnaires. This file contains Microsoft Words/PDF version of questionnaire statements used in the first and second round surveys (online).

**Additional file 2: Supplementary file 2.** GUIDED Checklist. This file contains GUIDED checklist used for reporting HDP pathways as the interventions developed in this study for improving HDP management in Indonesian primary care.

**Additional file 3: Supplementary file 3.** Statements that had not achieved consensus at the first and second-round survey, and the final agreement scores for each of the statements in the survey. This file contains two tables: Table 1. Statements that had not achieved consensus at the first and second-round survey, and Table 2. Final agreement scores for all statements tested in the survey.

## Data Availability

Supplementary files of the statements tested in the questionnaire and final agreement scores of the HDP statements are included in this publication. However, raw qualitative data and survey response materials are not shared due to protect the participants’ confidentiality.

## Abbreviations

ANC: Antenatal care
HDP: Hypertensive disorders of pregnancy
GPs: General practitioners
IQR: Interquartile ranges
IVF: In-vitro Fertilisation
JKN: *Jaminan Kesehatan Nasional* (Indonesian public health insurance)
LMICs: Low-and middle-income countries
Puskesmas: *Pusat Kesehatan Masyarakat* (Public primary care clinic)
REDCap: Research electronic data capture
