# Supplementary material for: Hypertensive disorders of pregnancy (HDP) management pathways: results of a Delphi survey to contextualise international recommendations for Indonesian primary care settings
Source: BMC Pregnancy Childbirth. 2021 Apr 1;21:269. doi: 10.1186/s12884-021-03735-3 (PMC8017638; doi:10.1186/s12884-021-03735-3)
Supplement: Supplementary file 1 — Additional file 1: Supplementary file 1. Survey questionnaires. This file contains Microsoft Words/PDF version of questionnaire statements used in the first and second round surveys (online). [file 12884_2021_3735_MOESM1_ESM.docx]

**Supplementary file 1. Microsoft words version of questionnaire statements used in the first and second round surveys (online)**

# First-round survey

## Opening questions:

1. **In your own words, how important are general practitioners (GPs) and other primary care providers’ (such as midwives and nurses) in the management of hypertension disorders of pregnancy (HDP)?**

**Answers:**

1. **In your own words, what are the potential roles that primary care providers can play in HDP management in primary care?**

**Answers:**

1. **Please write down below, if you think there are any enabling factors or challenges to managing hypertension in pregnancy in the Indonesian primary care.**

**Answers:**

## HDP Statements

The following statements on definitions, risk-factors, screening, diagnosis, prevention, and long-term follow-up of women with HDP in primary care were developed from our review of international guidelines and results from our exploratory interviews with primary care stakeholders in Indonesia. Your opinions and views on each statement are very important to us. They will help us to know whether the statements are applicable to HDP management in Indonesian primary care.

Please consider each statement in the context of:

- whether the statement contributes to improving care for pregnant women with high blood pressure and/or preeclampsia?
- whether the statement is applicable to Indonesian primary care settings?

Please rate each statement by clicking on the tick boxes, where 1 indicates that you strongly disagree with the statement and 5 indicates that you strongly agree with the statement.

| No | Recommendation | Ratings | | | | |
| --- | --- | --- | --- | --- | --- | --- |
|  |  | 1  Strongly disagree | 2 | 3 | 4 | 5  Strongly agree |
| Definition | | | | | | |
|  | Definition of HDP involves these descriptions:  a. Chronic hypertension is defined as high blood pressure (systolic blood pressure (SBP) ≥ 140mmHg or diastolic blood pressure (DBP) ≥ 90 mmHg) that is detected before the 20^th^ week of pregnancy. |  |  |  |  |  |
|  | b. Gestational hypertension is defined when the high blood pressure is first detected after 20^th^ week of pregnancy. |  |  |  |  |  |
|  | c. Masked hypertension is defined when normal blood pressure at the clinics, but it is elevated at other times. |  |  |  |  |  |
|  | d. Blood pressure measurement for patients suspected with masked hypertension is validated through ambulatory blood pressure monitoring or automated home blood pressure monitoring. |  |  |  |  |  |
|  | e. Transient hypertension is defined when high blood pressure is first detected at the 2^nd^ or 3^rd^ trimester, but it decreases through repeated measurements. |  |  |  |  |  |
|  | f. White coat hypertension is defined when patients have high blood pressure at clinic settings but decreases when they are away from the clinics (<135/85). |  |  |  |  |  |
|  | g. Preeclampsia is defined when high blood pressure (in more than the 20^th^ week) is accompanied by proteinuria or other organ abnormality or evidence of a small fetus. |  |  |  |  |  |
|  | All pregnant women should be screened for preeclampsia risk factors, such as:  High risk: previous history of preeclampsia. |  |  |  |  |  |
| Risk factors | |  |  |  |  |  |
|  | High risk: previous history of gestational hypertension |  |  |  |  |  |
|  | High risk: chronic hypertension |  |  |  |  |  |
|  | High risk: gestational diabetes |  |  |  |  |  |
|  | High risk: chronic diabetes mellites |  |  |  |  |  |
|  | High risk: chronic kidney disease |  |  |  |  |  |
|  | High risk: previous history of autoimmune disease: antiphospholipid syndrome |  |  |  |  |  |
|  | High risk: previous history of autoimmune disease: systemic lupus erythematosus |  |  |  |  |  |
|  | Moderate risk: first pregnancy |  |  |  |  |  |
|  | Moderate risk: maternal age more than 40 years or older |  |  |  |  |  |
|  | Moderate risk: receiving assisted reproduction |  |  |  |  |  |
|  | Moderate risk: twin pregnancy |  |  |  |  |  |
|  | Moderate risk: pregnancy with an interval of more than ten years |  |  |  |  |  |
|  | Moderate risk: BMI at the first visit >35 kg/m^2^ |  |  |  |  |  |
|  | Moderate risk: family history of preeclampsia |  |  |  |  |  |
|  | Moderate risk: SBP ≥ 130 mmHg at the first antenatal visit |  |  |  |  |  |
| Screening and diagnosis | |  |  |  |  |  |
|  | All pregnant women are encouraged to attend antenatal care (ANC) at a minimum of eight times during pregnancy. |  |  |  |  |  |
|  | All pregnant women should attend at least one integrated ANC in public primary care clinics (Puskesmas). |  |  |  |  |  |
|  | A minimum of one consultation with obstetrician should occur during the first two trimesters for women with HDP history. |  |  |  |  |  |
|  | The women’s blood pressure is measured at each antenatal visit. |  |  |  |  |  |
|  | Dipstick test is performed at least once in each trimester for all pregnant women. |  |  |  |  |  |
|  | Once the dipstick test is positive 1 (+1), the woman should be checked for other preeclampsia blood indicators. |  |  |  |  |  |
|  | Laboratory examination to confirm preeclampsia diagnosis, such as: kidney function test |  |  |  |  |  |
|  | Laboratory examination to confirm preeclampsia diagnosis, such as: liver function test |  |  |  |  |  |
|  | Laboratory examination to confirm preeclampsia diagnosis, such as: complete blood count |  |  |  |  |  |
|  | Examination performed to confirm preeclampsia, such as: fundal height measurement |  |  |  |  |  |
|  | Baseline reference for pregnant women with preeclampsia risk factors or in the area with high preeclampsia prevalence: haemoglobin. |  |  |  |  |  |
|  | Baseline reference for pregnant women with preeclampsia risk factors or in the area with high preeclampsia prevalence: platelet count. |  |  |  |  |  |
|  | Baseline reference for pregnant women with preeclampsia risk factors or in the area with high preeclampsia prevalence: serum creatinine. |  |  |  |  |  |
|  | Baseline reference for pregnant women with preeclampsia risk factors or in the area with high preeclampsia prevalence: uric acid. |  |  |  |  |  |
|  | Baseline reference for pregnant women with preeclampsia risk factors or in the area with high preeclampsia prevalence: urine protein. |  |  |  |  |  |
|  | GPs or midwives should refer a woman for an ultrasound if there is a possibility of fetal growth restriction following manual (fundal height) measurement. |  |  |  |  |  |
| Prevention | |  |  |  |  |  |
|  | All pregnant women are recommended to appropriately exercise during pregnancy to maintain their health. |  |  |  |  |  |
|  | Low dose aspirin is prescribed for women with one high risk or two moderate risk factors of preeclampsia from the 12-16^th^ week of pregnancy until the baby’s delivery. |  |  |  |  |  |
|  | Aspirin 75-150 mg is given daily at bedtime. |  |  |  |  |  |
|  | Pregnant women with risks of HDP with low daily calcium intake should also be offered with calcium supplementation 1.2-2.5 g/day. |  |  |  |  |  |
|  | A minimum dose of 500-600mg calcium per day is prescribed for women with low calcium intake. |  |  |  |  |  |
|  | Calcium supplementation is consumed at a different time to iron supplement, for example, iron in the morning and calcium in the evening. |  |  |  |  |  |
|  | All pregnant women have to be appropriately informed of their risks of developing preeclampsia. |  |  |  |  |  |
|  | GPs can prescribe low dose aspirin as preeclampsia prophylaxis. |  |  |  |  |  |
|  | GPs and midwives can prescribe calcium supplementation as prophylaxis of preeclampsia for women with low calcium intake. |  |  |  |  |  |
|  | GPs and midwives advise women with preeclampsia risks to regularly take aspirin and calcium supplementation. |  |  |  |  |  |
| Long-term follow up | |  |  |  |  |  |
|  | *Most antihypertensive agents can be used to control the women's blood pressure during breastfeeding periods*. |  |  |  |  |  |
|  | All women with HDP should be reviewed within three months after delivery. |  |  |  |  |  |
|  | HDP should resolve within three months after delivery. |  |  |  |  |  |
|  | If any abnormalities persist beyond the three months, women with a history of HDP should be referred to hospital. |  |  |  |  |  |
|  | Women with a history of HDP are recommended to achieve pre-pregnancy weight by 12 months after delivery. |  |  |  |  |  |
|  | Women with a history of HDP should receive postpartum counseling about their risks of cardiovascular diseases in the future. |  |  |  |  |  |
|  | Whenever appropriate, psychological counseling is given for women with a history of HDP |  |  |  |  |  |
|  | Healthy lifestyle counselling is given for women with a HDP history during postpartum periods. |  |  |  |  |  |
|  | Women with a history of HDP are counseled about postpartum contraception, aiming to provide adequate recovery time before conceiving again. |  |  |  |  |  |
|  | Any contraception methods can be prescribed for women with a history of HDP. |  |  |  |  |  |
|  | Non-hormonal contraception, such as IUD, is a priority for women with poor blood pressure control. |  |  |  |  |  |
|  | Women with a history of HDP should be prescribed with low dose aspirin for the next pregnancy. |  |  |  |  |  |
|  | Important health information about HDP should be noted at the women’s pregnancy book. |  |  |  |  |  |
|  | If you have any further suggestions or recommendation related to the definitions, risk-factors, screening, diagnosis, prevention or long-term follow up of hypertensive disorders of pregnancy, including preeclampsia and eclampsia, in primary care please list below (optional): | | | | | |

# Second-round survey

The following statements on management, monitoring, facilities and surveillance of women with HDP in primary care were developed from our review of international guidelines and results from exploratory interviews with primary care stakeholders in Indonesia. Your opinions and views on each statement are very important to us. They will help us to know whether the statements are applicable to HDP management in Indonesian primary care.

Please consider each statement in the context of:

- whether the statement contributes to improving care for pregnant women with high blood pressure and/or preeclampsia?
- whether the statement is applicable to Indonesian primary care settings?

Please rate each statement by clicking on the tick boxes, where 1 indicates that you strongly disagree with the statement and 5 indicates that you strongly agree with the statement.

| No | Recommendation | Ratings | | | | |
| --- | --- | --- | --- | --- | --- | --- |
|  |  | 1  Strongly disagree | 2 | 3 | 4 | 5  Strongly agree |
| HDP management in primary care | |  |  |  |  |  |
|  | Pregnant women should be consulted to an obstetrician once they are identified of having high blood pressure. |  |  |  |  |  |
|  | Antihypertensive agents should be considered once the women’s systolic blood pressure (SBP) >150 and/ or diastolic blood pressure (SBP) >100 mmHg. |  |  |  |  |  |
|  | Antihypertensive agents should be prescribed immediately once the women’s SBP ≥ 160 or DBP ≥ 110mmHg. |  |  |  |  |  |
|  | Regardless of the HDP diagnosis, blood pressure consistently over than 140/90 mmHg should be treated. |  |  |  |  |  |
|  | Antihypertensive medication given in primary care setting: Methyldopa. |  |  |  |  |  |
|  | Antihypertensive medication given in primary care setting: nifedipine. |  |  |  |  |  |
|  | Antihypertensive medication given in primary care setting: oral labetalol. |  |  |  |  |  |
|  | Pregnant women diagnosed with preeclampsia should be: referred to hospital. |  |  |  |  |  |
|  | Pregnant women diagnosed with preeclampsia should be: managed as an inpatient in a hospital. |  |  |  |  |  |
|  | Pregnant women diagnosed with preeclampsia should be: treated with magnesium sulphate injection as seizure prophylaxis. |  |  |  |  |  |
|  | In an emergency situation, primary care providers should give an initial dose of magnesium sulphate as a treatment for eclampsia seizures. |  |  |  |  |  |
|  | Consultation or telephone communication with obstetrician about preeclampsia treatment should be made prior to referral to the hospital. |  |  |  |  |  |
|  | Women with preeclampsia should be transported in an ambulance that has trained health care personnel and basic life support equipment. |  |  |  |  |  |
|  | Obstetrician-led delivery is arranged once pregnant women are diagnosed with HDP. |  |  |  |  |  |
|  | In the event that distance between the women’s residence to the hospital is far, pregnant women with pre-eclampsia are advised to re-locate immediately to areas near the hospital (e.g., waiting for a house^[[1]](#footnote-1)^ or a relative’s house^[[2]](#endnote-1)^). |  |  |  |  |  |
|  | Transportation to the referral centre or waiting house should be provided by family or community. |  |  |  |  |  |
| HDP monitoring in primary care | |  |  |  |  |  |
|  | Pregnant women with HDP are encouraged to attend hospital for their monitoring. |  |  |  |  |  |
|  | Pregnant women with HDP should monitor their blood pressure twice a week. |  |  |  |  |  |
|  | Primary care providers encourage women with HDP to be able to self-monitor their blood pressure. |  |  |  |  |  |
|  | Women with HDP should be monitored for signs and symptoms of preeclampsia, using evidence-based diagnostic tests. |  |  |  |  |  |
|  | Women with preeclampsia can be managed as an outpatient once the condition is stable. |  |  |  |  |  |
|  | Women with preeclampsia can be managed as an outpatient if they can be relied upon to reported problems. |  |  |  |  |  |
|  | Strict bedrest is not prescribed for women with HDP. |  |  |  |  |  |
|  | Salt restriction diet should not be prescribed for women with HDP. |  |  |  |  |  |
|  | Women with HDP must be referred to the hospital for a delivery plan before the 34^th^ week of pregnancy. |  |  |  |  |  |
|  | The induction of labour for women with non-complicated hypertension is planned at the 37^th^ week. |  |  |  |  |  |
|  | Delivery for women with severe and unstable preeclampsia should be conducted no later than the 34^th^ week of pregnancy. |  |  |  |  |  |
|  | Delivery for women with severe and unstable preeclampsia is conducted based on the consultation with an obstetrician. |  |  |  |  |  |
|  | All women with HDP should be encouraged to deliver their babies in a hospital with advanced obstetric and neonatal care supports. |  |  |  |  |  |
|  | Women with HDP can deliver their babies vaginally unless other complications arise that require further intervention. |  |  |  |  |  |
|  | The use of methyl ergonovine during labour and delivery for women with preeclampsia should be avoided. |  |  |  |  |  |
|  | The use of NSAIDs such as aspirin, ibuprofen, and diclofenac should be avoided for women with preeclampsia complicated with acute kidney injury during labour and delivery. |  |  |  |  |  |
|  | Blood pressure in women with HDP should be recorded shortly after birth-and if normal, should be checked again within 6 hours. |  |  |  |  |  |
|  | All women with a history of HDP and their babies should: be monitored. |  |  |  |  |  |
|  | All women with a history of HDP and their babies should: stay in hospital at least 24 hours postpartum. |  |  |  |  |  |
|  | All women with a history of HDP and their babies should: stay in the hospital until both are stable after delivery. |  |  |  |  |  |
|  | Postpartum follow up for women with preeclampsia are conducted during 24 hours after birth. |  |  |  |  |  |
|  | Postpartum follow up for women with preeclampsia are conducted during 48 hours after birth. |  |  |  |  |  |
|  | Postpartum follow up for women with preeclampsia are conducted during 72 hours after birth. |  |  |  |  |  |
|  | Postpartum follow up for women with preeclampsia are conducted for two weeks postpartum. |  |  |  |  |  |
|  | Postpartum follow up for women with preeclampsia are conducted for six weeks postpartum. |  |  |  |  |  |
|  | All women with HDP should be reminded of the warning signs and symptoms of preeclampsia following birth. |  |  |  |  |  |
|  | Preeclampsia blood examination for women with HDP needs to be repeated twice in the week after delivery. |  |  |  |  |  |
|  | Antihypertensive treatment prescribed for women with HDP should be continued in postpartum periods. |  |  |  |  |  |
|  | Antihypertensive treatment for women with HDP may be withdrawn carefully once their BP returns to normal levels. |  |  |  |  |  |
| Facilities | | | | | | |
|  | Medication and facilities for HDP management should be available in primary care practice: blood pressure measurement. |  |  |  |  |  |
|  | Medication and facilities for HDP management should be available in primary care practice: low dose aspirin-for preeclampsia prophylaxis. |  |  |  |  |  |
|  | Medication and facilities for HDP management should be available in primary care practice: antihypertensive medication. |  |  |  |  |  |
|  | Medication and facilities for HDP management should be available in primary care practice: magnesium sulphate injection. |  |  |  |  |  |
|  | Medication and facilities for HDP management should be available in primary care practice: calcium gluconate. |  |  |  |  |  |
|  | Medication and facilities for HDP management should be available in primary care practice: basic life supports, including oxygen and IV line. |  |  |  |  |  |
|  | HDP Guidelines should be available to inform nurses and midwives what to do for patient management. |  |  |  |  |  |
|  | Guidelines for magnesium sulphate injection should be available for nurses and midwives in an emergency situation. |  |  |  |  |  |
| Surveillance | |  |  |  |  |  |
|  | Private midwives/doctors’ practices should inform public primary care clinics (Puskesmas) once they identify a woman with HDP. |  |  |  |  |  |
|  | Public primary care clinics (Puskesmas) should follow up the patient by sending a cadre^[[3]](#endnote-2)^ to assist the woman during pregnancy. |  |  |  |  |  |
|  | Home visits to the HDP women’s house should be offered to: gain more information about the women’s condition. |  |  |  |  |  |
|  | Home visits to the HDP women’s house should be offered to: educate the women and her family member about the danger symptoms of preeclampsia. |  |  |  |  |  |
|  | Home visits to the HDP women’s house should be offered to: advise the women and their family about the importance of visiting health care provider during pregnancy. |  |  |  |  |  |
|  | Home visits to the women’s house are recommended within the first week after the woman discharged from the hospital. |  |  |  |  |  |
|  | If women with HDP give birth at the hospital, a copy of follow up or re-referral letter with the details of the delivery and follow up plans should be sent to the woman’s GP practice or public primary care (Puskesmas). |  |  |  |  |  |
|  | The cadre or community health workers should be involved in the postpartum monitoring plans for woman with HDP. |  |  |  |  |  |
|  | The cadre or community health workers should remind women with HDP to attend postpartum check at a hospital or GP’s practices. |  |  |  |  |  |
|  | Health promotion to prevent adolescent pregnancy be conducted as part of HDP prevention in the community. |  |  |  |  |  |
|  | If you have any further suggestions or recommendation related to the management, monitoring, facilities and surveillance of women with hypertensive disorders of pregnancy, including preeclampsia and eclampsia in primary care, please list below (optional): |  |  |  |  |  |

1. Often, distance between the women houses and hospitals in developing countries are far. A waiting house is a temporary house for women with high risks pregnancy but do not have any complication yet that enable them to live near the hospital for regular checks and visits at the hospital. [↑](#footnote-ref-1)
2. [↑](#endnote-ref-1)
3. In Indonesia, there are usually voluntary community health workers-or are also known as cadres, to help GPs and midwives working in the communities and transfer health information to pregnant women and their families. [↑](#endnote-ref-2)
