# Supplementary material for: Hypertensive disorders of pregnancy (HDP) management pathways: results of a Delphi survey to contextualise international recommendations for Indonesian primary care settings
Source: BMC Pregnancy Childbirth. 2021 Apr 1;21:269. doi: 10.1186/s12884-021-03735-3 (PMC8017638; doi:10.1186/s12884-021-03735-3)
Supplement: Supplementary file 3 — Additional file 3: Supplementary file 3. Statements that had not achieved consensus at the first and second-round survey, and the final agreement scores for each of the statements in the survey. This file contains two tables: Table 1. Statements that had not achieved consensus at the first and second-round survey, and Table 2. Final agreement scores for all statements tested in the survey. [file 12884_2021_3735_MOESM3_ESM.docx]

Supplementary file 3. Statements that had not achieved consensus at the first and second-round survey, and the final agreement score for each statement.

Table 1. Statements that had not reached consensus at the first and second-round survey^[[1]](#footnote-1)^.

| **No** | **Recommendation** | **Agreement score (%)** | **Quartile 1 (25^th^ percentile)** | **Median** | **Quartile 3 (75^th^ percentile)** | **Interquartile ranges**  **(IQR)** | **Standard Deviation (±)** |
| --- | --- | --- | --- | --- | --- | --- | --- |
|  | ***Statements that had not reached consensus at the first-round*** |  |  |  |  |  |  |
|  | *High-risk preeclampsia: previous history of systemic lupus erythematosus.* | *66.7* | *3.0* | 4.0 | 4.0 | *1.0* | *0.9* |
|  | *Moderate risk preeclampsia: receiving assisted reproduction.* | *56.3* | *3.0* | 4.0 | 4.0 | *1.0* | *1.0* |
|  | *These tests are recommended as baseline references for women with a high risk of preeclampsia:* |  |  |  |  |  |  |
|  | *Platelet count* | *66.7* | *3.0* | 4.0 | 5.0 | *2.0* | *1.1* |
|  | *Creatinine* | *68.8* | *3.0* | 4.0 | 4.3 | *1.3* | *1.0* |
|  | *Uric acid* | *47.9* | *2.8* | 3.0 | 4.0 | *1.3* | *1.1* |
|  | *Aspirin 75-150mg is given daily at bedtime.* | *68.8* | *3.0* | 4.0 | 4.0 | *1.0* | *0.8* |
|  | *GPs can prescribe low dose aspirin as prophylaxis for preeclampsia.* | *66.7* | *3.0* | 4.0 | 4.0 | *1.0* | *0.9* |
|  | *Most antihypertensive agents can be used to control the women's blood pressure during breastfeeding periods.* | *41.7* | *2.0* | 3.0 | 4.0 | *2.0* | *1.1* |
|  | *Any contraception methods can be prescribed for women with a history of HDP.* | *39.6* | *2.0* | 3.0 | 4.0 | *2.0* | *1.2* |
|  | *Women with a history of HDP should be prescribed with low dose aspirin for the next pregnancy.* | *58.3* | *3.0* | 4.0 | 5.0 | *1.0* | *0.9* |
|  | ***Statements that had not reached consensus at the second-round*** |  |  |  |  |  |  |
|  | *These following medications can be given for women with HDP in primary care settings:* |  |  |  |  |  |  |
|  | -        *Methyldopa* | *60.0* | 3.0 | 4.0 | 4.0 | *1.0* | *1.0* |
|  | -        *Labetalol* | *48.9* | 3.0 | 4.0 | 4.0 | *1.0* | *1.1* |
|  | *Strict bed rest is not prescribed for women with HDP.* | *62.2* | 3.0 | 4.0 | 4.0 | *1.0* | *0.9* |
|  | *Salt restriction diet should not be prescribed for women with HDP.* | *33.3* | 2.0 | 3.0 | 4.0 | *2.0* | *1.0* |
|  | *The use of NSAIDs such as aspirin, ibuprofen, and diclofenac should be avoided for women with preeclampsia in labour and delivery.* | *57.8* | 3.0 | 4.0 | 4.0 | *1.0* | *1.0* |

Table 2. Final agreement scores for each statement

| **No** | **Recommendation** | **Final agreement score (%)** | **Quartile 1 (25^th^ percentile)** | **Median** | **Quartile 3**  **(75^th^ percentile)** | **Interquartile ranges**  **(IQR)** | **Standard Deviation (±)** |  |
| --- | --- | --- | --- | --- | --- | --- | --- | --- |
| **First-round survey statements** | | | | | | | | |
|  | Definition of HDP involves these descriptions: |  |  |  |  |  |  |  |
| 1. | a. Chronic hypertension is defined as high blood pressure (systolic blood pressure (SBP) ≥ 140mmHg or diastolic blood pressure (DBP) ≥ 90 mmHg) that is detected before the 20^th^ week of pregnancy. | 87.5 | 4.0 | 4.0 | 5.0 | 1.0 | 1.0 |  |
| 2. |  | 87.5 |  |  |  | 1.0 | 0.9 |  |
|  | b. Gestational hypertension is defined when the high blood pressure is first detected after 20^th^ week of pregnancy. |  | 4.0 | 4.0 | 5.0 |  |  |  |
| 3. | c. Masked hypertension is defined when normal blood pressure at the clinics, but it is elevated at other times. | 85.4 | 4.0 | 4.0 | 4.0 | 0.0 | 0.8 |  |
| 4. | d. Blood pressure measurement for patients suspected with masked hypertension is validated through ambulatory blood pressure monitoring or automated home blood pressure monitoring. | 91.7 | 4.0 | 4.0 | 5.0 | 1.0 | 0.7 |  |
| 5. | e. Transient hypertension is defined when high blood pressure is first detected at the 2^nd^ or 3^rd^ trimester, but it decreases through repeated measurements. | 75.0 | 3.8 | 4.0 | 4.0 | 0.3 | 0.9 |  |
| 6. | f. White coat hypertension is defined when patients have high blood pressure at clinic settings but decreases when they are away from the clinics (<135/85). | 87.5 | 4.0 | 4.0 | 5.0 | 1.0 | 0.8 |  |
| 7. | g. Preeclampsia is defined when high blood pressure (in more than the 20^th^ week) is accompanied by proteinuria or other organ abnormality or evidence of a small foetus. | 91.7 | 4.0 | 4.0 | 5.0 | 1.0 | 0.8 |  |
|  | **Mean agreement score on definition statements** | 86.6 | 4.0 | 4.0 | 4.7 | 0.7 | 0.8 |  |
| 8. | All pregnant women should be screened for preeclampsia risk factors, such as: | 87.5 | 4.0 | 4.0 | 5.0 | 1.0 | 0.5 |  |
| 8.  9. | High risk: previous history of preeclampsia. | 87.5  87.5 | 4.0 | 4.0 | 5.0 | 1.0  1.0 | 0.5  0.7 |  |
|  | High risk: previous history of gestational hypertension |  | 4.0 | 4.0 | 5.0 |  |  |  |
| 10. | High risk: chronic hypertension | 85.4 | 4.0 | 4.0 | 5.0 | 1.0 | 0.7 |  |
| 11. | High risk: gestational diabetes | 91.7 | 4.0 | 4.0 | 5.0 | 1.0 | 0.7 |  |
| 12. | High risk: chronic diabetes mellites | 75.0 | 4.0 | 4.0 | 4.3 | 0.3 | 0.8 |  |
| 13. | High risk: chronic kidney disease | 87.5 | 4.0 | 4.0 | 5.0 | 1.0 | 0.7 |  |
| 14. | High risk: previous history of autoimmune disease: antiphospholipid syndrome | 91.7 | 4.0 | 4.0 | 5.0 | 1.0 | 0.8 |  |
| 15. | High risk: previous history of autoimmune disease: systemic lupus erythematosus | 72.9 | 3.0 | 4.0 | 4.3 | 1.3 | 0.9 |  |
| 16. | Moderate risk: first pregnancy | 75.0 | 3.8 | 4.0 | 5.0 | 1.3 | 1.1 |  |
| 17. | Moderate risk: maternal age more than 40 years or older | 97.9 | 4.0 | 4.0 | 5.0 | 1.0 | 0.5 |  |
| 18. | Moderate risk: receiving assisted reproduction*^[[2]](#footnote-2)^ | *56.3* | *3.0* | *4.0* | *4.0* | *1.0* | *1.0* |  |
| 19. | Moderate risk: twin pregnancy | 83.3 | 4.0 | 4.0 | 4.3 | 0.3 | 0.8 |  |
| 20. | Moderate risk: pregnancy with an interval of more than ten years | 77.1 | 4.0 | 4.0 | 4.3 | 0.3 | 0.9 |  |
| 21. | Moderate risk: BMI at the first visit >35 kg/m2 | 89.6 | 4.0 | 4.0 | 4.0 | 0.0 | 0.6 |  |
| 22. | Moderate risk: family history of preeclampsia | 81.3 | 4.0 | 4.0 | 4.3 | 0.3 | 0.9 |  |
| 23. | Moderate risk: SBP ≥ 130 mmHg at the first antenatal visit | 70.8 | 3.0 | 4.0 | 4.0 | 1.0 | 0.8 |  |
|  | **Mean agreement score on risk factor statements** | 81.9 | 3.8 | 4.0 | 4.6 | 0.8 | 0.8 |  |
| 24. | All pregnant women are encouraged to attend antenatal care (ANC) at a minimum of eight times during pregnancy. | 75.0 | 3.8 | 4.0 | 4.0 | 0.3 | 1.0 |  |
| 25. | All pregnant women should attend at least one integrated ANC in public primary care clinics (Puskesmas)^[[3]](#footnote-3)^. | 77.1 | 4.0 | 4.0 | 5.0 | 1.0 | 1.2 |  |
| 26. | A minimum of one consultation with obstetrician should occur during the first two trimesters for women with HDP history. | 87.5 | 4.0 | 4.0 | 5.0 | 1.0 | 0.8 |  |
| 27. | The women’s blood pressure is measured at each antenatal visit. | 100.0 | 4.0 | 5.0 | 5.0 | 1.0 | 0.5 |  |
| 28. | Dipstick test is performed at least once in each trimester for all pregnant women. | 70.8 | 3.0 | 4.0 | 4.0 | 1.0 | 1.0 |  |
| 29. | Once the dipstick test is positive 1 (+1), the woman should be checked for other preeclampsia blood indicators. | 89.6 | 4.0 | 4.0 | 5.0 | 1.0 | 0.8 |  |
| **30.** | Laboratory examination to confirm preeclampsia diagnosis, such as: kidney function test | 95.8 | 4.0 | 4.0 | 5.0 | 1.0 | 0.7 |  |
| 31. | Laboratory examination to confirm preeclampsia diagnosis, such as: liver function test | 79.2 | 4.0 | 4.0 | 5.0 | 1.0 | 0.9 |  |
| 32. | Laboratory examination to confirm preeclampsia diagnosis, such as: complete blood count | 81.3 | 4.0 | 4.0 | 5.0 | 1.0 | 0.9 |  |
| 33. | Laboratory examination to confirm preeclampsia diagnosis, such as: fundal height measurement | 70.8 | 3.0 | 4.0 | 4.0 | 1.0 | 0.9 |  |
| 34. | Baseline reference for pregnant women with preeclampsia risk factors or in the area with high preeclampsia prevalence: haemoglobin. | 95.8 | 4.0 | 5.0 | 5.0 | 1.0 | 0.7 |  |
| 35. | Baseline reference for pregnant women with preeclampsia risk factors or in the area with high preeclampsia prevalence: platelet count. | 70.8 | 3.0 | 4.0 | 4.0 | 1.0 | 1.0 |  |
| 36. | Baseline reference for pregnant women with preeclampsia risk factors or in the area with high preeclampsia prevalence: serum creatinine. | 70.8 | 3.0 | 4.0 | 5.0 | 2.0 | 1.0 |  |
| 37. | *Baseline reference for pregnant women with preeclampsia risk* factors *or in the area with high preeclampsia prevalence: uric acid.* | *52.1* | *3.0* | *4.0* | *4.0* | *1.0* | *1.0* |  |
| 38. | Baseline reference for pregnant women with preeclampsia risk factors or in the area with high preeclampsia prevalence: urine protein. | 93.2 | 4.0 | 4.0 | 5.0 | 1.0 | 0.7 |  |
| 39. | GPs or midwives should refer a woman for an ultrasound if there is a possibility of foetal growth restriction following manual (fundal height) measurement. | 100.0 | 4.0 | 4.0 | 5.0 | 1.0 | 0.5 |  |
|  | **Mean agreement score on screening and diagnosis statements** | 81.9 | 3.7 | 4.1 | 4.7 | 1.0 | 0.8 |  |
| 40. | All pregnant women are recommended to appropriately exercise during pregnancy to maintain their health. | 91.7 | 4.0 | 4.0 | 5.0 | 1.0 | 0.8 |  |
| 41. | Low dose aspirin is prescribed for women with one high risk or two moderate risk factors of preeclampsia from the 12-16^th^ week of pregnancy until the baby’s delivery. | 77.1 | 4.0 | 4.0 | 4.0 | 0.0 | 0.7 |  |
| 42. | Aspirin 75-150 mg is given daily at bedtime. | 70.8 | 3.0 | 4.0 | 4.0 | 1.0 | 0.8 |  |
| 43. | Pregnant women with risks of HDP with low daily calcium intake should also be offered with calcium supplementation 1.2-2.5 g/day. | 77.1 | 4.0 | 4.0 | 4.0 | 0.0 | 0.6 |  |
| 44. | A minimum dose of 500-600mg calcium per day is prescribed for women with low calcium intake. | 79.2 | 4.0 | 4.0 | 4.0 | 0.0 | 0.8 |  |
| 45. | Calcium supplementation is consumed at a different time to iron supplement, for example, iron in the morning and calcium in the evening. | 79.2 | 4.0 | 4.0 | 5.0 | 1.0 | 0.8 |  |
| 46. | All pregnant women have to be appropriately informed of their risks of developing preeclampsia. | 97.9 | 4.0 | 4.0 | 5.0 | 1.0 | 0.6 |  |
| 47. | GPs can prescribe low dose aspirin as preeclampsia prophylaxis. | 70.8 | 3.0 | 4.0 | 4.0 | 1.0 | 0.9 |  |
| 48. | GPs and midwives can prescribe calcium supplementation as prophylaxis of preeclampsia for women with low calcium intake. | 81.3 | 4.0 | 4.0 | 4.0 | 0.0 | 0.8 |  |
| 49. | GPs and midwives advise women with preeclampsia risks to regularly take aspirin and calcium supplementation. | 85.4 | 4.0 | 4.0 | 5.0 | 1.0 | 0.8 |  |
|  | **Mean agreement score on prevention statements** | 81.1 | 3.8 | 4.0 | 4.4 | 0.6 | 0.8 |  |
| 50. | *Most antihypertensive agents can be used to control the women's blood pressure during breastfeeding periods *.* | *47.9* | *2.0* | *3.0* | *4.0* | *2.0* | *1.1* |  |
| 51. | All women with HDP should be reviewed within three months after delivery. | 79.2 | 4.0 | 4.0 | 5.0 | 1.0 | 0.9 |  |
| 52. | HDP should resolve within three months after delivery. | 85.4 | 4.0 | 4.0 | 5.0 | 1.0 | 0.7 |  |
| 53. | If any abnormalities persist beyond the three months, women with a history of HDP should be referred to hospital. | 91.7 | 4.0 | 4.0 | 5.0 | 1.0 | 0.7 |  |
| 54. | Women with a history of HDP are recommended to achieve pre-pregnancy weight by 12 months after delivery. | 79.2 | 4.0 | 4.0 | 4.0 | 0.0 | 0.7 |  |
| 55. | Women with a history of HDP should receive postpartum counselling about their risks of cardiovascular diseases in the future. | 100.0 | 4.0 | 4.0 | 5.0 | 1.0 | 0.5 |  |
| 56. | Whenever appropriate, psychological counselling is given for women with a history of HDP | 93.8 | 4.0 | 4.0 | 5.0 | 1.0 | 0.7 |  |
| 57. | Healthy lifestyle counselling is given for women with a HDP history during postpartum periods. | 97.9 | 4.0 | 4.0 | 5.0 | 1.0 | 0.5 |  |
| 58. | Women with a history of HDP are counselled about postpartum contraception, aiming to provide adequate recovery time before conceiving again. | 100.0 | 4.0 | 4.0 | 5.0 | 1.0 | 0.5 |  |
| 59. | *Any contraception methods can be prescribed for women with a history of HDP**. | 37.5 | 2.0 | 3.0 | 4.0 | 2.0 | 1.3 |  |
| 60. | Non-hormonal contraception, such as IUD, is a priority for women with poor blood pressure control. | 95.8 | 4.0 | 4.0 | 5.0 | 1.0 | 0.5 |  |
| 61. | *Women with a history of HDP should be prescribed with low dose aspirin for the next pregnancy*. | 64.6 | 3.0 | 4.0 | 4.0 | 1.0 | 0.8 |  |
| 62. | Important health information about HDP should be noted at the women’s pregnancy book. | 100.0 | 4.0 | 4.5 | 5.0 | 1.0 | 0.5 |  |
|  | **Mean agreement score on long-term follow up in primary care statements** | 82.5 | 3.6 | 3.9 | 4.7 | 1.1 | 0.7 |  |
| **Second-round survey statements** | | | | | | | |  |
| 63. | Pregnant women should be consulted to an obstetrician once they are identified of having high blood pressure. | 95.6 | 4.0 | 4.0 | 5.0 | 1.0 | 0.7 |  |
| 64. | Antihypertensive agents should be considered once the women’s systolic blood pressure (SBP) >150 and/ or diastolic blood pressure (SBP) >100 mmHg. | 82.9 | 4.0 | 4.0 | 5.0 | 1.0 | 0.9 |  |
| 65. | Antihypertensive agents should be prescribed immediately once the women’s SBP ≥ 160 or DBP ≥ 110mmHg. | 94.0 | 4.0 | 4.0 | 5.0 | 1.0 | 0.8 |  |
| 66. | Regardless of the HDP diagnosis, blood pressure consistently over than 140/90 mmHg should be treated. | 77.8 | 4.0 | 4.0 | 5.0 | 1.0 | 1.0 |  |
| 67. | *Antihypertensive medication given in primary care setting:*  m*ethyldopa.* | *60.0* | *3.0* | *4.0* | *4.0* | *1.0* | *1.0* |  |
| 68. | Antihypertensive medication given in primary care setting: nifedipine. | 88.9 | 4.0 | 4.0 | 5.0 | 1.0 | 0.8 |  |
| 69. | *Antihypertensive medication given in primary care setting: oral labetalol.* | *48.9* | *3.0* | *4.0* | *4.0* | *1.0* | *1.1* |  |
| 70. | Pregnant women diagnosed with preeclampsia should be referred to hospital. | 93.3 | 4.0 | 5.0 | 5.0 | 1.0 | 0.6 |  |
| 71. | Pregnant women diagnosed with preeclampsia should be managed as an inpatient in a hospital. | 73.3 | 3.0 | 4.0 | 5.0 | 2.0 | 1.0 |  |
| 72. | Pregnant women diagnosed with preeclampsia should be treated with magnesium sulphate injection as seizure prophylaxis. | 77.8 | 4.0 | 4.0 | 5.0 | 1.0 | 0.9 |  |
| 73. | In an emergency situation, primary care providers should give an initial dose of magnesium sulphate as a treatment for eclampsia seizures. | 88.9 | 4.0 | 4.0 | 5.0 | 1.0 | 0.7 |  |
| 74. | Consultation or telephone communication with obstetrician about preeclampsia treatment should be made prior to referral to the hospital. | 80.0 | 4.0 | 4.0 | 5.0 | 1.0 | 0.8 |  |
| 75. | Women with preeclampsia should be transported in an ambulance that has trained health care personnel and basic life support equipment. | 95.6 | 4.0 | 5.0 | 5.0 | 1.0 | 0.7 |  |
| 76. | Obstetrician-led delivery is arranged once pregnant women are diagnosed with HDP. | 95.6 | 4.0 | 4.0 | 5.0 | 1.0 | 0.6 |  |
| 77. | In the event that distance between the women’s residence to the hospital is far, pregnant women with pre-eclampsia are advised to re-locate immediately to areas near the hospital (e.g., waiting for a house or a relative’s house^[[4]](#footnote-4)^. | 88.9 | 4.0 | 4.0 | 5.0 | 1.0 | 0.7 |  |
| 78. | Transportation to the referral centre or waiting house should be provided by family or community. | 86.7 | 4.0 | 4.0 | 5.0 | 1.0 | 0.7 |  |
|  | **Mean agreement scores on management statements** | 83.0 | 3.8 | 4.1 | 4.9 | 1.1 | 0.8 |  |
| 79. | Pregnant women with HDP are encouraged to attend hospital for their monitoring. | 84.4 | 4.0 | 4.0 | 5.0 | 1.0 | 0.9 |  |
| 80. | Pregnant women with HDP should monitor their blood pressure twice a week. | 73.3 | 3.0 | 4.0 | 4.0 | 1.0 | 1.0 |  |
| 81. | Primary care providers encourage women with HDP to be able to self-monitor their blood pressure. | 88.9 | 4.0 | 4.0 | 5.0 | 1.0 | 0.7 |  |
| 82. | Women with HDP should be monitored for signs and symptoms of preeclampsia, using evidence-based diagnostic tests. | 97.8 | 4.0 | 4.0 | 5.0 | 1.0 | 0.5 |  |
| 83. | Women with preeclampsia can be managed as an outpatient once the condition is stable. | 82.2 | 4.0 | 4.0 | 4.0 | 0.0 | 0.8 |  |
| 84. | Women with preeclampsia can be managed as an outpatient if they can be relied upon to reported problems. | 73.3 | 3.0 | 4.0 | 4.0 | 1.0 | 0.9 |  |
| 85. | *Strict bedrest is not prescribed for women with HDP.* | *62.2* | *3.0* | *4.0* | *4.0* | *1.0* | *0.9* |  |
| 86. | *Salt restriction diet should not be prescribed for women with HDP.* | *33.3* | *2.0* | *3.0* | *4.0* | *2.0* | *1.0* |  |
| 87. | Women with HDP must be referred to the hospital for a delivery plan before the 34^th^ week of pregnancy. | 82.2 | 4.0 | 4.0 | 4.0 | 0.0 | 0.9 |  |
| 88. | The induction of labour for women with non-complicated hypertension is planned at the 37^th^ week. | 71.1 | 3.0 | 4.0 | 4.0 | 1.0 | 1.0 |  |
| 89. | Delivery for women with severe and unstable preeclampsia should be conducted no later than the 34^th^ week of pregnancy. | 75.6 | 4.0 | 4.0 | 4.0 | 0.0 | 1.0 |  |
| 90. | Delivery for women with severe and unstable preeclampsia is conducted based on the consultation with an obstetrician. | 100.0 | 4.0 | 5.0 | 5.0 | 1.0 | 0.5 |  |
| 91. | All women with HDP should be encouraged to deliver their babies in a hospital with advanced obstetric and neonatal care supports. | 88.9 | 4.0 | 4.0 | 5.0 | 1.0 | 0.8 |  |
| 92. | Women with HDP can deliver their babies vaginally unless other complications arise that require further intervention. | 93.3 | 4.0 | 4.0 | 5.0 | 1.0 | 0.5 |  |
| 93. | The use of methyl ergonovine during labour and delivery for women with preeclampsia should be avoided. | 80.0 | 4.0 | 4.0 | 4.0 | 0.0 | 0.7 |  |
| 94. | *The use of NSAIDs such as aspirin, ibuprofen, and diclofenac should be avoided for women with preeclampsia complicated with acute kidney injury during labour and delivery.* | *57.8* | *3.0* | *4.0* | *4.0* | *1.0* | *1.0* |  |
| 95. | Blood pressure in women with HDP should be recorded shortly after birth-and if normal, should be checked again within 6 hours. | 86.7 | 4.0 | 4.0 | 4.0 | 0.0 | 0.8 |  |
| 96. | All women with a history of HDP and their babies should: be monitored. | 100.0 | 4.0 | 4.0 | 5.0 | 1.0 | 0.5 |  |
| 97. | All women with a history of HDP and their babies should: stay in hospital at least 24 hours postpartum. | 97.8 | 4.0 | 4.0 | 5.0 | 1.0 | 0.6 |  |
| 98. | All women with a history of HDP and their babies should: stay in the hospital until both are stable after delivery. | 91.1 | 4.0 | 4.0 | 5.0 | 1.0 | 0.7 |  |
| 99. | Postpartum follow up for women with preeclampsia are conducted during 24 hours after birth. | 95.6 | 4.0 | 4.0 | 5.0 | 1.0 | 0.6 |  |
| 100. | Postpartum follow up for women with preeclampsia are conducted during 48 hours after birth. | 97.8 | 4.0 | 4.0 | 5.0 | 1.0 | 0.5 |  |
| 101. | Postpartum follow up for women with preeclampsia are conducted during 72 hours after birth. | 93.3 | 4.0 | 4.0 | 5.0 | 1.0 | 0.6 |  |
| 102. | Postpartum follow up for women with preeclampsia are conducted for two weeks postpartum. | 93.3 | 4.0 | 4.0 | 5.0 | 1.0 | 0.6 |  |
| 103. | Postpartum follow up for women with preeclampsia are conducted for six weeks postpartum. | 88.9 | 4.0 | 4.0 | 5.0 | 1.0 | 0.7 |  |
| 104. | All women with HDP should be reminded of the warning signs and symptoms of preeclampsia following birth. | 95.6 | 4.0 | 5.0 | 5.0 | 1.0 | 0.7 |  |
| 105. | Preeclampsia blood examination for women with HDP needs to be repeated twice in the week after delivery. | 86.7 | 4.0 | 4.0 | 5.0 | 1.0 | 0.8 |  |
| 106. | Antihypertensive treatment prescribed for women with HDP should be continued in postpartum periods. | 73.3 | 3.0 | 4.0 | 5.0 | 2.0 | 0.8 |  |
| 107. | Antihypertensive treatment for women with HDP may be withdrawn carefully once their BP returns to normal levels. | 95.6 | 4.0 | 4.0 | 5.0 | 1.0 | 0.5 |  |
|  | **Mean agreement score on monitoring statements** | 84.1 | 3.7 | 4.0 | 4.6 | 0.9 | 0.7 |  |
| 108. | Medication and facilities for HDP management should be available in primary care practice: blood pressure measurement. | 100.0 | 4.0 | 5.0 | 5.0 | 1.0 | 0.4 |  |
| 109. | Medication and facilities for HDP management should be available in primary care practice: low dose aspirin-for preeclampsia prophylaxis. | 93.3 | 4.0 | 5.0 | 5.0 | 1.0 | 0.6 |  |
| 110. | Medication and facilities for HDP management should be available in primary care practice: antihypertensive medication. | 97.8 | 4.0 | 5.0 | 5.0 | 1.0 | 0.5 |  |
| 111. | Medication and facilities for HDP management should be available in primary care practice: magnesium sulphate injection. | 95.6 | 4.0 | 5.0 | 5.0 | 1.0 | 0.6 |  |
| 112. | Medication and facilities for HDP management should be available in primary care practice: calcium gluconate. | 93.3 | 4.0 | 5.0 | 5.0 | 1.0 | 0.6 |  |
| 113. | Medication and facilities for HDP management should be available in primary care practice: basic life supports, including oxygen and IV line. | 100.0 | 4.0 | 5.0 | 5.0 | 1.0 | 0.5 |  |
| 114. | HDP Guidelines should be available to inform nurses and midwives what to do for patient management. | 100.0 | 4.0 | 5.0 | 5.0 | 1.0 | 0.5 |  |
| 115. | Guidelines for magnesium sulphate injection should be available for nurses and midwives in an emergency situation. | 95.6 | 4.0 | 5.0 | 5.0 | 1.0 | 0.6 |  |
|  | **Mean agreement score on facility statements** | 96.9 | 4.0 | 5.0 | 5.0 | 1.0 | 0.5 |  |
| 116. | Private midwives/doctors’ practices should inform public primary care clinics (Puskesmas) once they identify a woman with HDP. | 97.8 | 4.0 | 5.0 | 5.0 | 1.0 | 0.5 |  |
| 117. | Public primary care clinics (Puskesmas) should follow up the patient by sending a cadre^[[5]](#footnote-5)^ to assist the woman during pregnancy. | 97.8 | 4.0 | 4.0 | 5.0 | 1.0 | 0.5 |  |
| 118. | Home visits to the HDP women’s house should be offered to: gain more information about the women’s condition. | 100.0 | 4.0 | 5.0 | 5.0 | 1.0 | 0.5 |  |
| 119. | Home visits to the HDP women’s house should be offered to: educate the women and her family member about the danger symptoms of preeclampsia. | 100.0 | 4.0 | 5.0 | 5.0 | 1.0 | 0.5 |  |
| 120. | Home visits to the HDP women’s house should be offered to: advise the women and their family about the importance of visiting health care provider during pregnancy. | 97.8 | 4.0 | 4.0 | 5.0 | 1.0 | 0.5 |  |
| 121. | Home visits to the women’s house are recommended within the first week after the woman discharged from the hospital. | 97.8 | 4.0 | 4.0 | 5.0 | 1.0 | 0.6 |  |
| 122. | If women with HDP give birth at the hospital, a copy of follow up or re-referral letter with the details of the delivery and follow up plan should be sent to the woman’s GP practice or public primary care (Puskesmas). | 100.0 | 4.0 | 5.0 | 5.0 | 1.0 | 0.5 |  |
| 123. | The cadre or community health workers should be involved in the postpartum monitoring plans for woman with HDP. | 97.8 | 4.0 | 4.0 | 5.0 | 1.0 | 0.5 |  |
| 124. | The cadre or community health workers should remind women with HDP to attend postpartum check at a hospital or GP’s practices. | 100.0 | 4.0 | 4.0 | 5.0 | 1.0 | 0.5 |  |
| 125. | Health promotion to prevent adolescent pregnancy be conducted as part of HDP prevention in the community. | 91.1 | 4.0 | 5.0 | 5.0 | 1.0 | 0.7 |  |
|  | **Mean agreement score on HDP surveillance statements** | 98.0 | 4.0 | 4.5 | 5.0 | 1.0 | 0.5 |  |
| **Third-round survey (pathways agreement)** | | | | | | | |  |
| 1 | **Hypertensive disorders of pregnancy (HDP) diagnosis flowchart.** | 86.5 | 4.0 | 4.0 | 4.0 | 0.0 | 0.8 |  |
| 2 | Hypertensive disorders of pregnancy (HDP) management pathways in primary care: (i) screening for preeclampsia risk factors. | 89.2 | 4.0 | 4.0 | 5.0 | 1.0 | 0.9 |  |
| 3 | Hypertensive disorders of pregnancy (HDP) management pathways in primary care: (ii) HDP screening during routine ANC. | 81.1 | 4.0 | 4.0 | 5.0 | 1.0 | 1.0 |  |
| 4 | Hypertensive disorders of pregnancy (HDP) management pathways in primary care: (iii) HDP management and monitoring. | 78.4 | 4.0 | 4.0 | 5.0 | 1.0 | 1.1 |  |
| 5 | Hypertensive disorders of pregnancy (HDP) management pathways in primary care: (iv) delivery planning for women with HDP. | 86.5 | 4.0 | 4.0 | 5.0 | 1.0 | 0.9 |  |
| 6 | Hypertensive disorders of pregnancy (HDP) management pathways in primary care: (v) postpartum follow up for women with HDP. | 83.8 | 4.0 | 4.0 | 5.0 | 1.0 | 0.9 |  |
|  | **Mean agreement score for HDP management pathways** | 83.8 | 4.0 | 4.0 | 5.0 | 1.0 | 1.0 |  |
| 7 | **Surveillance pathway for women with HDP in primary care.** | 86.5 | 4.0 | 4.0 | 5.0 | 1.0 | 0.8 |  |

1. Five-point Likert scale used in the study: 1= strong disagreement, 2 = disagreement, 3 = indicated neutral position, 4 = agreement, and 5 = strong agreement. [↑](#footnote-ref-1)
2. Statements written in italic were those that did not reach consensus at the survey. Statements written in italic and have * were not retested at the third-round survey. [↑](#footnote-ref-2)
3. Public primary care clinic in Indonesia is also known as Puskesmas [↑](#footnote-ref-3)
4. Often, distance between women’s houses and hospitals in Indonesia is far. A waiting house is a temporary house for the women to enable them stay close to the hospital for pregnancy monitoring. [↑](#footnote-ref-4)
5. Community health workers are also known as cadres. [↑](#footnote-ref-5)
